# Photo-induced macro/mesoscopic scale ion displacement in mixed-halide perovskites: ring structures and ionic plasma oscillations

**DOI:** 10.1038/s41377-022-00957-8

**Published:** 2022-09-07

**Authors:** Xiaoxiao Sun, Yong Zhang, Weikun Ge

**Affiliations:** 1 Laboratory for Thin Films and Photovoltaics, Empa−Swiss Federal Laboratories for Materials Science and Technology, 8600 Duebendorf, Switzerland; 2 Department of Information Technology and Electrical Engineering, ETH Zurich, 8093 Zurich, Switzerland; 3 Institute of Ion Beam Physics and Materials Research, Helmholtz-Zentrum Dresden-Rossendorf, Dresden, 01328 Germany; 4 Department of Electrical and Computer Engineering, The University of North Carolina at Charlotte, Charlotte, NC 28223 USA; 5 Department of Physics, Tsinghua University, Beijing, 10084 People’s Republic of China

**Keywords:** Optical physics, Optical materials and structures

## Abstract

Contrary to the common belief that the light-induced halide ion segregation in a mixed halide alloy occurs within the illuminated area, we find that the Br ions released by light are expelled from the illuminated area, which generates a macro/mesoscopic size anion ring surrounding the illuminated area, exhibiting a photoluminescence ring. This intriguing phenomenon can be explained as resulting from two counter-balancing effects: the outward diffusion of the light-induced free Br ions and the Coulombic force between the anion deficit and surplus region. Right after removing the illumination, the macro/mesoscopic scale ion displacement results in a built-in voltage of about 0.4 V between the ring and the center. Then, the displaced anions return to the illuminated area, and the restoring force leads to a damped ultra-low-frequency oscillatory ion motion, with a period of about 20–30 h and lasting over 100 h. This finding may be the first observation of an ionic plasma oscillation in solids. Our understanding and controlling the “ion segregation” demonstrate that it is possible to turn this commonly viewed “adverse phenomenon” into novel electronic applications, such as ionic patterning, self-destructive memory, and energy storage.

## Abstract (web summary)

Contrary to the commonly assumed local segregation in mixed halide perovskite alloys, this work reveals the nonlocal nature of the “segregation”, a macroscopic/mesoscopic scale oscillatory charge displacement.

## Graphical abstract

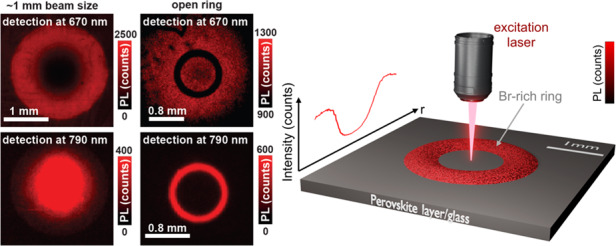

## Introduction

Lead halide perovskites (e.g., MAPbI_3_) are an emerging family of semiconductor materials with excellent optoelectronic properties ideally suited for photovoltaic and light-emitting applications^1–3^. This class of soft crystals is known to be mixed conductors of both electronic and ionic conductivity. Significant ion migration has been reported in these materials and is one of the main mechanisms responsible for anomalous I-V hysteresis and poor stability in the perovskite solar cells^4–6^. Particularly, as initially reported in 2015^7^, mixed halide perovskites (e.g., MAPbI_1-*x*_Br_*x*_) further exhibit photoinduced halide anion “segregation” under continuous above-bandgap illumination, and the process is reversible when the illumination is removed^7–11^. This segregation phenomenon is commonly viewed as an adverse effect on optoelectronic applications and should be suppressed^12^. The most commonly observed effect of the ion segregation is the redshift in photoluminescence (PL) peak from the expected wavelength for the alloy to that of a significantly higher I composition^13^. It is generally asserted that a uniform alloy MAPbI_1-*x*_Br_*x*_ would segregate into Iodide-rich and Bromide-rich domains within the illuminated area. A number of microscopic mechanisms have been proposed to explain the phenomenon^14–18^. They can be summarized into three categories:^13^ (1) Thermodynamic in origin; (2) polaron-induced lattice strain; (3) photogenerated carrier gradient or electric field, interacting with point defects. However, none of them can unambiguously explain all the key aspects of the phenomenon^13^. In fact, the chemical and structural characteristics of the so-called “Br-rich” and “I-rich” regions are not yet well understood, although they are implicitly assumed to be simply Br-rich and I-rich alloys, respectively.

Regardless of which model was invoked, it was always taken for granted that the segregation occurred within the illuminated area and implicitly assumed that there was no change in the overall cation-anion stoichiometry other than the local exchange of the anions in a similar manner as in the composition modulation found in other semiconductor alloys. Composition modulation is commonly referred to as the nonuniform spatial distribution of the chemical elements deviating from the expected value of the alloy composition in a length scale beyond the unit cell, usually occurring spontaneously during the growth^19^. Note that previous studies were typically performed under uniform illumination over a macroscopic sample area. Spatially resolved PL or cathodoluminescence (CL) probe within the illuminated area revealed that the conversion was nonuniform, but the overall area of the “I-rich” regions increased with illumination time, and eventually appeared to reach a steady state covering the entire illuminated area^15,20,21^. Besides the most readily observable PL peak shift, other circumstantial evidence for the ion segregation includes split XRD peaks^7^, below bandgap optical absorption^16^, etc. However, there is no direct evidence, such as spatially resolved elementary analysis, for ion segregation. It has been noted that even in the fully converted state, the “I-rich” regions only represent a small fraction of the overall volume, based on the optical absorption strength and XRD intensity of the “I-rich” regions^7,8,20^. If the Br ions remained in the illuminated area, as implied, the complete quenching of the alloy state PL would suggest that the “I-rich” regions were uniformly embedded within the “Br-rich” matrix with an average separation distance shorter than the carrier diffusion length of the “Br-rich” matrix. Since the carrier diffusion lengths in such polycrystalline films are relatively short, typically in the order of μm^22,23^ and comparable to the polycrystalline domain sizes, the average separation of the “I-rich” regions is expected to be rather small. It was suggested that the sizes of the “I-rich” regions were sub-10 nm^15,21^ and near the surface^20,21^. In short, previous efforts have focused on the microscopic scale anion segregation within the illuminated area under uniform light illumination.

In stark contrast, we discover that the anion segregation in the mixed halide alloys is a nonlocal effect of which the ion redistribution may occur in a macroscopic or mesoscopic scale beyond but proportional to the illumination beam size up to well over mm. Specifically, we find that under illumination, within the illumination area, the PL peak is red shifting from the initial position; while concurrently, outside the illuminated area, the alloy PL peak is strongly enhanced in a ring area circling the illuminated area. Furthermore, the process is reversible but non-monotonically, exhibiting ultra-low-frequency damped oscillations between the ring and center in terms of PL intensity and position. These surprising observations can be explained as that free Br ions are expelled from the illuminated area, resulting in a positively charged area, and concomitantly forming a negatively charged Br-rich ring, both being off stoichiometry from the original alloy. This phenomenon can be viewed as an ionic analogy of a mesoscopic PL ring formation away from the illuminated site in GaAs/AlGaAs quantum wells, resulting from the disparity in the electron and hole diffusion lengths and thus their spatial profiles^24–27^. For the latter, ionic motion is irrelevant. However, in the current case, the ionic diffusion plays the dominant role, whereas the carrier diffusion is negligible in the relevant length scale. The peculiar oscillatory behavior could reflect an oscillation of ionic plasma, which has not been reported in solids and has no electronic equivalence. Besides their own significance, these new findings offer new insights into the underlying mechanism of the ion segregation in the mixed halide alloys, which makes it not necessarily an adverse phenomenon to suppress but something potentially useful, e.g., for energy storage.

## Results

### Forming a remote ring-like structure modification

“Pump-probe” type experiments are performed on polycrystalline mixed-halide MA_0.17_FA_0.83_Pb(I_0.5_Br_0.5_)_3_ thin films on glass substrates. Different from the common “pump-probe” experiment, we probe the sample well beyond the action time and physical scale of the “pump beam”. The sample is first illuminated (“pumped”) locally with a 639 nm laser beam, with the beam size varying from 1 μm to 1 mm, to induce the halide ion redistribution; then, the slow dynamics of the ionic motion are probed over an area much beyond the “pump” beam size by spatially resolved continuous-wave (CW) PL mapping with the same laser wavelength (typically at reduced power) as a function of time, up to 130 h after the illumination. The dynamics are also probed by time-resolved (TR) PL mapping and CW absorption after the illumination. Details about the samples and measurements are given in Methods.

**Fig. 1:**
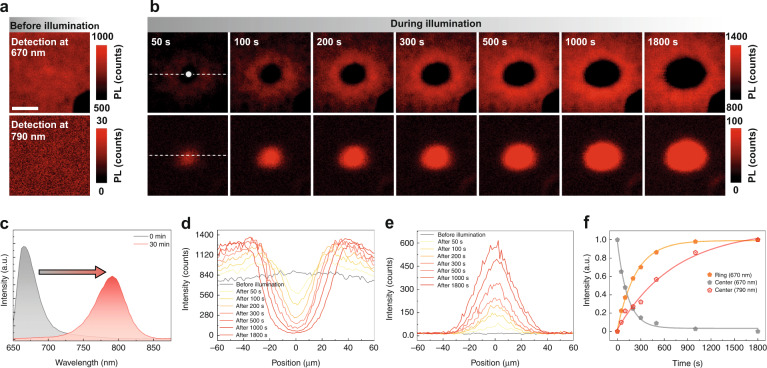
Time evolution of the light-induced spatial variation in PL for a MA_0.17_FA_0.83_Pb(I_0.5_Br_0.5_)_3_ thin-film sample. **a** PL mapping at 670 and 790 nm before local illumination (The dark area at the lower right corner is due to a marker). The scale bar is 40 μm. **b** PL mapping at 670 nm (top row) and 790 nm (bottom row) of the same area as in (**a**) after the center of the area being illuminated for different effective illumination times. The small dot indicates the pump beam size. **c** PL spectra at the initial state and after illumination over 30 min at the center. **d**, **e** Time-dependent PL spatial profiles taken along the white dashed lines from PL maps of 670 and 790 nm in (**b**), respectively. **f** The normalized time dependence of 670 nm PL intensity at the ring and center from (**d**), and 790 nm PL intensity at the center from (**e**). Symbols are data points, and lines are fitting curves

Figure 1 shows the time evolution of the light-induced spatial variation in PL that reflects the halide ion redistributions over a mesoscopic scale of about ten times the illumination beam size (∼12 μm). Firstly, as typically done in the literature, PL spectra are measured from the illuminated site at different illumination times over 30 min at 0.1 W cm^−2^ (see Supplementary Fig. S1), which yields the expected results: the quenching of the alloy PL peak at ∼670 nm for *x*_Br_ = 0.5, accompanying by a redshift of the overall emission band to ∼790 nm. This phenomenon has been attributed to light-induced halide ion segregation, with the latter peak being explained as the emission from “I-rich” regions^15,20,28–31^. Secondly, as references, confocal PL mapping is performed at 670 and 790 nm (in 10 nm bandwidth), respectively, over an area of 120 μm × 120 μm, on the as-grown sample under a probe power density of 0.3 W cm^−2^ and acquisition time of 10 ms/point (below 2 × 10^−5^ of the conversion time), as shown in Fig. 1a. The probe beam will not affect the sample during PL mapping with the short acquisition time (see Methods for detailed information). Then, the sample is illuminated at the center of the mapped area with the same beam size at a power density of 1.5 W cm^−2^. By briefly shutting off the pump beam at different effective illumination times, the effects of the ion redistribution caused by the pump beam are probed by performing speedy PL mapping at 670 and 790 nm over the extended area (see Methods for the implementation and the definition of the effective illumination time). As shown by the results in Fig. 1b, upon the local illumination is turned on, a dark disk starts to form in the 670 nm PL mapping, while a complementary and comparable size bright disk appears in the 790 nm mapping, and the size and contrast of the two disks increase with the illumination time. More interestingly, in the meantime, a bright ring of the 670 nm emission emerges and establishes outside the illuminated area, resulting in a donut shape intensity distribution. Figure 1c shows the spectra of the initial state and after 30 min illumination time at the center, which is qualitatively similar to Supplementary Fig. S1, except for some subtle changes in the relative intensity between 670 and 790 nm emission.

This surprising observation suggests a new perspective of the ion transport in the halide alloy: light-induced ion diffusion from illuminated into non-illuminated region instead of local segregation. It is reasonable to conclude that some free Br ions are expelled from the illuminated region, but there seems to exist a balancing force preventing them from either simply dispersing into a practically infinitely large area surrounding the illuminated region, i.e., forming a diffusion-length-limited decaying profile, as in the case of outward carrier diffusion under local excitation typically seen in a semiconductor^32–34^. Following the commonly accepted understanding, one would conclude that the emergence of the 790 nm emission at the illuminated region was simply due to the formation of small I-rich domains embedded in a Br-rich matrix, and nothing would happen outside the illuminated area. However, the formation of the ring exterior of the illuminated area suggests a very different scenario: the local illumination generates free Br ions in the illuminated region, the concentration gradient leads to the outward diffusion of the free Br ions through halide vacancies, and the resulting electric field due to the charge imbalance establishes a finite diffusion boundary, resembling the formation of a p-n junction in a semiconductor. Further elaboration of this idea will be provided below. Since the Br depletion region grows with time to over four times in diameter of the pump beam size in this case, it may involve an impact ion releasing process along the diffusion path, because the diffusing ions from the center have kinetic energy and momentum. Importantly, the fact that the diffusion of the free Br ions into the otherwise non-perturbed area makes the PL emission there stronger suggests that the added ions passivate the halide vacancies in the ring area and leave behind more halide vacancies in the illuminated area and nearby.

To analyze the ring formation process more quantitatively, a series of line profiles corresponding to different local illumination times along the white dashed lines in Fig. 1b are plotted in Fig. 1d, e, respectively, for the 670 and 790 nm emission. At the center, the 670/790 nm intensity decreases/increases monotonically with time, and at the outer edge of the ring (approximately ±40 μm from the center), the 670 nm intensity also increases monotonically, but the intensity for a position in between exhibits a non-monotonical time dependence, indicating that the intermediate region is being refilled by the free Br ions from the center while in the meantime the free Br ions are moving outward from there. We further analyze the kinetics of the 670 nm emission intensity at the center (0 μm) and the edge of the ring (±40 μm), as plotted in Fig. 1f, by fitting the data to exponential dependences: $I_0 + \Delta I \ast e^{ - t/\tau }$ for the decay, and $I_0 + \Delta I \ast (1 - e^{ - t/\tau })$ for the enhancement (*I*_0_– initial intensity, $\Delta I -$ the magnitude of change). The fitting yields time constants $\tau _d^C$ (670 nm) = 132 s and $\tau _r^R$ (670 nm) = 238 s, corresponding to the decay at the center and enhancement at the ring, respectively. As expected, the depletion speed is faster than that of the ring formation because the latter is a convoluted process of the free ion generation at the illuminated area and outward diffusion to the ring. The time difference between the two processes is roughly the ion diffusion time. Also included in Fig. 1f is the time dependence of the 790 nm emission at the illumination site. The rise time of $\tau _r^C$ (790 nm) = 738 s is much longer than the decay time of $\tau _d^C$ (670 nm). However, the integrated PL longer than 675 nm at the illumination site from Supplementary Fig. S1 yields a rise time of $\tau _r^C$ (> 675 nm) = 186 s, nearly identical to the decay time of $\tau _d^C$ (<675 nm) = 192 s (Supplementary Fig. S2), which indicates that the illuminated site senses the effect of the Br ion releasing concurrently with 670 nm emission being quenched, although some structural relaxation time, a trickling down process, is needed to reach the stable configuration that yields 790 nm emission. This finding suggests that the appearance of the 790 nm emission does not require an ion exchange process for the I ions of the exterior of the illuminated site (e.g., the ring area) to refill the lost Br ions, which would take a long time since I ions are known to have smaller diffusivity^35–37^. This understanding is in stark contrast to the commonly accepted picture in which ion segregation does not change the local charge balance. This situation is also very different from the thermal-induced interdiffusion between Br-rich and I-rich regions, where a uniform alloy is achieved at the end and the process is irreversible^38,39^. Thus, the so-called “ion segregation” process is largely the escape of the more loosely bound Br ions, which might be located at some defect sites (e.g., the domain boundaries) initially, from the illuminated region. This understanding is consistent with the literature observation that the conversion occurs at the defects, particularly the grain boundaries, within the illuminated region^15,21,28^, but the destination of the released Br ions is totally different.

**Fig. 2:**
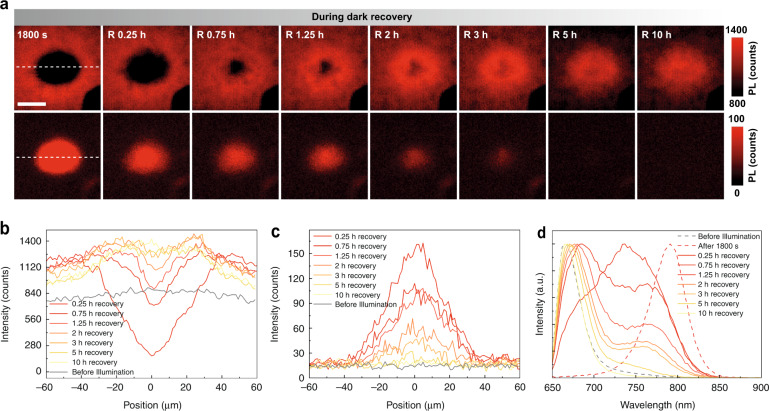
The recovery process of the light-induced ion redistribution. **a** PL mapping at different recovery times for 670 nm (top row) and 790 nm (bottom row). The scale bar is 40 μm. **b**, **c** Time-dependent PL spatial profiles taken at the white dashed lines in (**a**) for 670 and 790 nm, respectively. **d** Dynamic tracking of normalized PL spectra at the illuminated site

At the end of the study reported in Fig. 1, the center pump beam is turned off permanently, and the recovery kinetics of the extended area is monitored in the dark. Figure 2a summarized the speedy PL mapping with the probe power at different delay times. The 670 nm PL ring gradually shrinks to the center and eventually forms a disk at the center, showing about 50% higher intensity than the initial state. In the meantime, the 790 nm emission disk at the center fades away. The changes in intensity can be better seen in Fig. 2b, c for the line profiles along the dashed lines in Fig. 2a, respectively, for 670 and 790 nm, whereas the profiles before illumination are also included for comparison. During the recovery process, we also collect the PL spectra at the center, as shown in Fig. 2d. The comparison of the normalized spectra before illumination and after 10 h recovery show nearly the same peak wavelength but with slightly less tail emission. This indicates that the recovered state of the polycrystalline material is somewhat less defective because when the free Br ions return, they might find energetically more favorable sites to form a more ordered structure. The recovery process is much slower than the ring formation process, which can be explained by the fact that the formation process has an external driving force, whereas the recovery process mainly relies on the stored energy of the electric field created by the non-equilibrium Br ion distribution. This understanding is consistent with the oscillatory behavior to be discussed later. Furthermore, the processes only involve the lateral motions of the ions, because, for the sample studied here, the film thickness is comparable to the absorption length (~460 nm^40^), thus, there is no significant variation along the vertical direction. Indeed, similar PL mapping measurements from the back (glass) side show very similar results as from the top surface (see Supplementary Fig. S3). In some previous studies, because shorter wavelength lasers with shorter absorption depths were used, the effect was only observed near the surface, thus, the “ion segregation” was concluded to occur near the surface^20,21^.

**Fig. 3:**
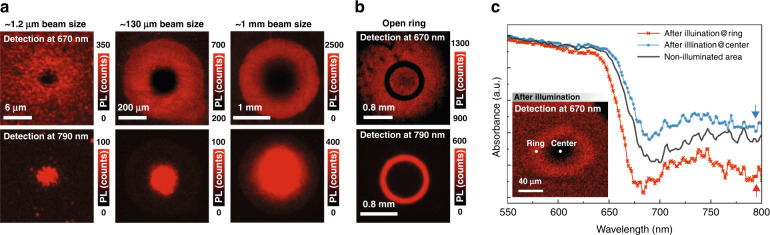
The ion distribution with different pump beam sizes and shapes and the chemical composition analysis of the ring structure. **a** PL emission mapping at a detection wavelength of 670 and 790 nm after local illumination for 1 h at beam size of ~1.2 μm/8 suns, ~130 μm/1 sun, and ~1 mm/1 sun, respectively. **b** PL mapping after local illumination with a beam size of ~1 mm over an annular aperture with a 1 mm diameter pinhole and a center obstruction target of 850 μm. **c** Absorption spectra of the film at the brightest position of the ring and the center of the illuminated region with a reference included. Inset shows PL mapping at 670 nm after local light illumination by ~12 μm beam for 1 h

The phenomena reported above are universal for the pump beam size down to 1 μm and up to 1 mm, as shown in Fig. 3a for the PL mapping results for a few additional beam sizes right after conversion. For the largest beam size, the exterior diameter of the Br-rich ring is as large as 2 mm, showing truly macroscopic scale ion diffusion. We also note that the above findings are qualitatively valid for different illumination power densities, for instance, similar results are also observed under the 1 sun equivalent illumination, except for the process being much slower. The PL emission mapping and time dependences of 670 and 790 nm emission at the illuminated site are shown for different beam sizes under the same power density (0.8 W/cm^2^) in Supplementary Fig. S4. The normalized decay and rise processes at the illuminated site are consistent for different beam sizes, except for the 670 nm curve with the smallest (~1.2 μm) beam size. Since this beam size is comparable to the polycrystalline domain size^16,20,29^, there might be more fluctuation (see different sets of 670 nm time map in Supplementary Fig. S5), while a larger beam size averages over many domains.

To further verify the above results are not artifacts caused by stray light, as well as examine the possibility of repelling the free Br ions from the illuminated site to more than just the outward direction, we perform the conversion measurements by placing an annular aperture (a 1 mm pinhole with a central obstruction target of 850 μm) on top of the sample, and then illuminated the sample for 1 h with a 1 mm beam at the center of the aperture. Figure 3b shows the 670 nm mapping with a dark ring matching the aperture open area, accompanying by a bright outer ring and a bright inner disk; and a complementary bright ring in the 790 nm mapping. These results suggest that the free Br ions can be expelled in any directions, either inward to the center or outward, by a concentration gradient. The recovery process is qualitatively similar to that shown in Fig. 2 (see Supplementary Fig. S6).

To gain more insight into the chemical compositions at the center and ring region, we performed in situ optical transmission measurements after local light illumination (see Methods for details). The sample is illuminated with ∼12 μm beam at 0.3 W cm^−2^ to generate the ring structure, with the effect shown in the inset of Fig. 3c for the 670 nm PL mapping. Figure 3c presents the typical absorption spectra from the brightest position of the ring and the center of the illuminated region, with a reference to the initial state. The absorption edge of the ring or center exhibits a moderate blue or redshift, suggesting some increase and decrease of the Br content, respectively, compared to the reference. However, the slight increase of the Br content in the ring might just result in the reduction of halide vacancies or improved crystallinity, as implied by the sharper absorption edge and reduced tail absorption. Accordingly, at the center, the red-shifted absorption edge and increased tail absorption suggest that the illuminated region becomes more defective, having more halide vacancies with slightly less Br content. The observed minor change in the illuminated region indicates that it has not been converted into an I-rich alloy, as commonly believed, which further precludes the possibility that significant I ions move into the illuminated region while free Br ions move out. Therefore, the 790 nm emission originated from the newly created defects in the alloy rather than the I-rich alloy domains. Along this line, the generation of the mobile ions can be understood as below. The as-grown film contains a large density of defects, particularly anion vacancies. Hence, some Br ions adjacent to the defect sites are only loosely bonded to the cations in the ground state, and they are typically associated with trapped states above the valance band. Upon photoexcitation, some electrons at these defective Br sites are excited, and the bonds are further weakened, such that these ions become mobile with a concentration proportional to the power density and irradiation time. These freed ions start to diffuse away from the illuminated site via vacancies due to the concentration gradient. However, only a portion of the free Br ions can actually diffuse away, because the recombination of the photoexcited carriers may re-anchor some disturbed free Br ions. Together with the Coulomb attractive force generated by the unbalanced cations at the illuminated site, the three processes jointly establish a steady-state spatial distribution of the free Br ions. This explanation is supported by the presence of a built-in potential of 0.4 ± 0.1 V between the center and ring measured right after removing the illumination (for details, see Supplementary Fig. S7). Assuming that the charge distributions in the illuminated and ring area are uniform, we provide a quantitative description of the built-in potential using the Gauss theorem:

1 $$V_{{\mathrm{build-in}}} = \frac{{qN_2}}{{2\varepsilon }}r_2^2ln\left(\frac{{r_2}}{{r_1}}\right)$$

where *N*_1_ is the density of the positive charges in the illuminated area, *N*_2_ is the density of negative charges (expelled free Br ions) in the ring area, and $N_2 = r_1^2N_1/(r_2^2 - r_1^2)$with *r*_1_ being the radius of the illuminated area and *r*_2_ is the outer radius of the ring. The ring structure parameters for the 1 mm beam size in Fig. 3 yield *N*_1_ = 4.8 × 10^9^ cm^−3^ and *N*_2_ = 1.25 × 10^9^ cm^−3^. Note that *N*_1_ represents the Br ion deficit in the illuminated area, while *N*_2_ the surplus in the ring area. During the local illumination, the free Br ion generation rate *G* can be approximated by the free electronic carrier generation rate by assuming that each valence electron being excited frees one Br ion; and each interband recombination of an electron-hole pair anchors down one Br ion. Under steady-state, the outward diffusion and the inverse drift process of the Br ions are balanced, then the free Br ions density in the illuminated area can be estimated by

2 $$N_{{\mathrm{free}}} = G \ast \tau$$

where *τ* is the carrier decay time. Under 1 sun with the measured average PL decay time ~200 ns, the estimated density of free Br ions is *N*_free_ ~ 1.4 × 10^15^ cm^−3^, which is comparable to the literature reported defect density in the film^41–43^, where the vacancies provide the paths for the ionic motion. The details of the calculations are provided in the Supplementary.

The defect origin of the 790 nm emission is supported by the observation that the relative intensity of the 790 and 670 nm emission is very sensitive to the excitation density (e.g., Fig. 1c at 1.5 W cm^−2^ vs. Supplementary Fig. S1 at 0.1 W cm^−2^). Because the defect sites are limited, the 790 nm emission tends to relatively weaken with increasing illumination density in our measurement, even diminish at further higher illumination density (e.g., 100 W/cm^2^ at 532 nm, although there the effect was explained by the polaron saturation)^44^.

### TRPL of the nonlocal “ion segregation”

**Fig. 4:**
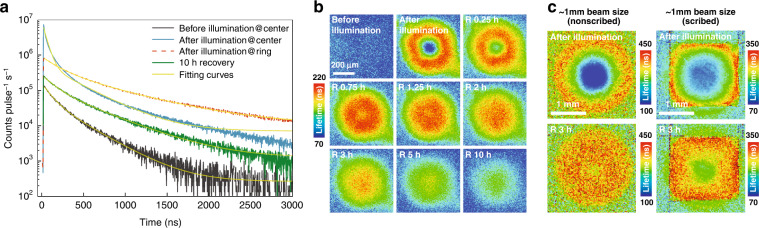
The dynamics of the ion redistribution probed by TRPL. **a** PL decay traces at different illumination states. **b** TRPL mapping before and after local illumination and during the recovery process. **c** TRPL mapping after local illumination in a non-scribed and scribed area

TRPL may provide further insight into the ion redistribution effects observed above in the CW measurements. We perform TRPL mapping before and after local illumination and monitor the recovery. The sample is first illuminated with 130 μm beam size and at 0.1 W cm^−2^ for 1 h to generate the ring structure (Fig. 3a). Figure 4a depicts a few key TRPL traces: before illumination, from the center and ring at the end of the illumination, and from the center after 10 h recovery (see Supplementary Fig. S8 for the complete series). The PL decay traces can be well fitted using a biexponential function $I\left( t \right) = A_1{\mathrm{exp}}\left( { - t/\tau _1} \right) + A_2{\mathrm{exp}}\left( { - t/\tau _2} \right)$, and an effective decay lifetime can be defined as $\tau _{{\mathrm{avg}}} = \frac{{A_1\tau _1^2 + A_2\tau _2^2}}{{A_1\tau _1 + A_2\tau _2}}$^45^. Figure 4b shows the mapping results of *τ*_avg_ before and after the ring formation, and at different recovery times. After illumination, at the ring area, the lifetimes are the longest and greatly increase from the initial state value, whereas at the center, the lifetime is the shortest, even below the initial state value. However, after 10 h recovering, the lifetime at the center becomes substantially longer than the initial state value. These findings are qualitatively consistent with the trends found in the CW results in Figs. 1, 2, and consistent with the ideas that after illumination, the ring area exhibits better crystallinity, and after recovery the center also becomes less defective.

One might wonder what will happen if the whole sample is illuminated, since many of the previous studies were performed in this way. To this end, we attempt to scribe the thin film to achieve an effective sample size comparable to the mm scale beam size, and perform the TRPL mapping after local illumination at the non-scribed and scribed area, respectively. The measurement condition is the same as that of Fig. 4b but with a larger beam size (~1 mm). Since the phenomena are universal for different beam sizes, Fig. 4c shows similar features of the TRPL mapping results after the ring formation and during recovery time (in the non-scribed area). However, for the scribed square area comparable to the beam size, the ring is truncated by the sample “edges” (the scribed lines) that limit the free Br ions diffusion. After 3 h recovering, the expelled free Br ions gradually return to the center from the “edges” of the sample. These findings further support our conclusion that the so-called “ion segregation” process is largely the motion of the released Br ions from the illuminated site. More scribing experiments on the “edge” effects are provided in Supplementary Figs. S9, 10.

### Ultra-low-frequency oscillations

**Fig. 5:**
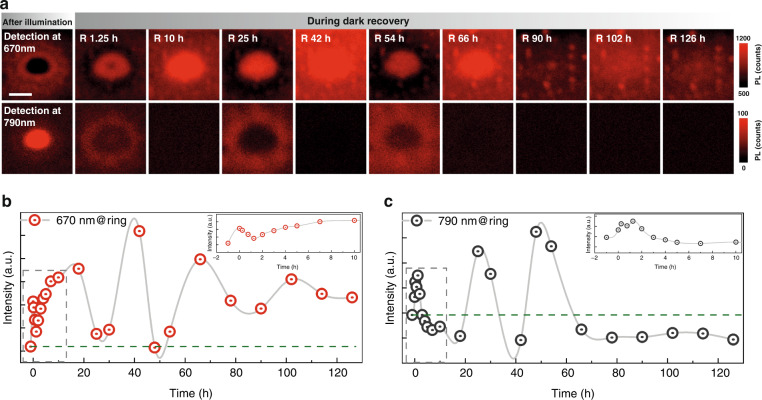
Oscillatory phenomenon of light-induced ion redistribution. **a** PL mapping after local illumination and at selected recovery times for 670 nm (top row) and 790 nm (bottom row). The scale bar is 40 μm. **b**, **c** Time-dependent PL intensity taken at the ring in (**a**) for 670 and 790 nm, respectively. Insets show a zoom-in view of the time range before 10 h

Using the measurement condition of Fig. 1, with a lower probe power density (0.1 W cm^−2^), by monitoring over a longer recovery time (>100 h), a striking new feature emerges: an oscillatory phenomenon is observed during recovery, as shown in Fig. 5a. In the upper row for the 670 nm PL mapping at selected times (see Supplementary Fig. S11 for the complete series), the ring size shrinks, and intensity oscillates between ring and center, while a complementary and periodic cycle appears in the 790 nm mapping. Since the “ion segregation” process is largely a redistribution of the free Br ions, the oscillatory behavior suggests that the motion of the free Br ions, an ionic plasma, has a restoring force that could be the Coulombic interaction and concentration gradient between the Br ion deficit center and surplus ring. We envision a process: under a local illumination, the photo-released free Br ions, like high viscosity liquid, are pushed away from the illuminated center; but when the perturbation stops, they will return with overshooting, due to jointly the restoring force and inverted concentration gradient; an oscillation will establish itself under suitable conditions. This picture is supported by the plots in Fig. 5b, c, the time-dependent PL intensities at the ring for 670 and 790 nm. The initial intensity is at the “negative” time, whereas *t* = 0 h is right after illumination. The green dashed lines show the initial state values. After removing the illumination, both 670 and 790 nm intensity oscillate with time, suggesting that the free Br ions behave like an ionic plasmon with a very low oscillation frequency because of the heavy ionic mass and screening. While the oscillations are eventually damped with time, they do not return to the original states before the illumination. Although we limit ourselves to a qualitative discussion here, we finish by noting that the observation of the ionic oscillation indicates that free Br ions can flow and oscillate like electronic plasma. Further investigation will be required to fully understand the dynamics of the oscillation system.

## Discussion

By showing the unambiguous presence of the nonlocal effects far beyond the illuminated site, this work offers a completely new perspective to the commonly assumed photoinduced “on-site” anion segregation in mixed halide perovskite alloys. We have provided the first demonstration of a PL ring structure, resulting from the disparity in the ionic distributions between halide anions and complementary cations in mixed halide alloys under local optical excitation, as an analogy of the electronic-based phenomenon in GaAs/AlGaAs QW systems. We have further observed an oscillatory behavior of the photogenerated free ions that could be the first reported ionic plasma oscillation in solids. Understanding and controlling the “ion segregation” phenomenon is now far beyond merely the goal of mitigating the adverse effects of solar cell applications. Instead, it will open up new avenues for investigating macroscopic or mesoscopic scale ion transport and the effects of ionic plasma in solids and applying the effects in next-generation electronics (e.g., ionic patterning, self-destructive memory, energy storage).

## Materials and methods

### Growth of perovskite films

Mixed-halide perovskite MA_0.17_FA_0.83_Pb(I_0.5_Br_0.5_)_3_ precursor solution was prepared by dissolving PbI_2_ (0.46 M), PbBr_2_ (0.86 M), formamidinium iodide (1 M), and methylammonium bromide (0.2 M) in a mixture of anhydrous DMF:DMSO (4:1 volume ratio, v-v). Glass substrates were cleaned by sonication in acetone and isopropyl alcohol, then the substrates were further cleaned with oxygen plasma treatment for 15 min. The perovskite solution was spin-coated on glass substrates in a two-step program at 1000 and 4000 rpm for 10 and 35 s, respectively, and 110 μL of chlorobenzene was poured on the spinning substrate 30 s after the starting of the program. The substrates were then annealed at 100 °C for 1 h. The 490 ± 10 nm thick films were deposited in a nitrogen glove box under moisture- and oxygen- controlled conditions. Fig. S12 in the supplementary information shows the X-ray diffraction (XRD) spectrum and atomic force microscopy (AFM) image of the film. A profilometer (AlphaStep P120) was used to get the film thickness. We first made a scratch by using a razor blade on the film and then probed the height difference between the glass and thin film. After growth, the sample was cut into small pieces, and stored in a glove box to prevent severe oxidation. Each experiment was then performed on a new piece of absorber, freshly removed from the nitrogen box. This allowed us to investigate a large number of different measurement conditions on the same sample from the same growth run.

The MAI and FABr were purchased from Greatcell Solar Materials (>99.99%), the lead salts from TCI (99.99%), DMSO (99.9%), DMF (99.8%), and chlorobenzene (99.8%) from Sigma-Aldrich.

### Spatially resolved CW PL mapping

A MicroTime 100 system coupled to a detection unit from PicoQuant was used for PL/TRPL mapping. A wide range scanner stage from Physik Instruments was used to locate the sample. For photon counting, PicoQuant TCSPC cards in the long-range mode were employed to avoid pile-up effects. Olympus objectives (with 4×/NA0.1, 20×/NA0.45, 100×/NA0.9) corresponding to different excitation beam sizes were used. For each lens, the system can operate in either confocal or widefield illumination mode, resulting in beam sizes from ∼1 μm to 1 mm. A detection fiber of 50 μm acting as a pinhole collects the light coming from the objective to the entrance of the monochromator. The PL image scans over an area of 10 μm × 10 μm to 2.5 mm × 2.5 mm, with (100 × 100) pixels, at each pixel, the dwelling time is between 5 to 10 ms.

The measurements performed in this work could be considered a typical form of “pump-probe” measurement. In contrast to the conventional pump-probe measurement, where one probes the effect of carriers generated by the pump beam within the illuminated area of the pump beam at different delay times, here we probe the dynamics of the ion redistribution generated by the “pump beam”, instead of carriers, over an area far beyond that illuminated by the pump beam, not only during the illumination process but also over a time scale 10–100 h after the pump beam is turned off. Because of the relatively short carrier lifetime (in the order of 100 ns) relative to the time scale of interest, the carriers generated by the pump beam do not have any significant impact on the dynamics.

For the results shown in Fig. 1b, the goal is to probe how a localized illumination can affect the material property over a spatial range much beyond the illuminated area (e.g., ten times the illumination beam size) at different continuous illumination times. Specifically, we need to measure the PL spectrum at each point from an area much larger (e.g., 10×) than the illuminated site under continuous illumination. This is not a trivial task. Conceptually, it can be achieved in such a way: a relatively high power (pump beam) is used for the local illumination to induce the material change; a lower power beam (probe beam), which will not induce any significant material change by itself during the probing time, is then used to probe the whole area of interest. A commercial spectroscopy system with such capability is not available. We have developed a scheme to perform this task using the existing optical system. The scheme is detailed below.

**Fig. 6:**
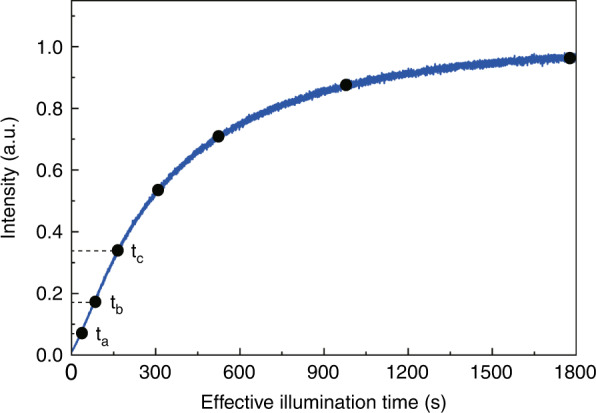
A calibration curve for pump and probe measurements. Normalized 790 nm PL intensity vs. illumination time

We first generated a calibration curve, the intensity of the 790 nm PL peak as a function of illumination time, as shown below in Fig. 6, measured with ∼12 μm beam size and 1.5 W cm^−2^ excitation density. We recorded the time sequences at a selected set of illumination times *t*_*a*_, *t*_*b*_, *t*_*c*_,…, and their intensities *I*_*a*_, *I*_*b*_, *I*_*c*_,…. We then selected a new location on the sample to obtain the data shown in Fig. 1b. Specifically, at time *t*_*a*_, the illumination was interrupted, the laser was switched to the probe power to perform a speedy PL mapping; then switched back to the pump power to illuminate the center again till the 790 nm PL intensity reaching the predetermined value *I*_*b*_, and stopped the illumination again, performed the same PL mapping; etc. Therefore, the effective illumination time is the time required to reach a predetermined state if the illumination was continuously applied. Note that the conversion process is a relatively slow and cumulative process, and the recovery process is even slower. If we make a very short break during the illumination process and resume the illumination again, the impact will be minimal. Our system can only perform a point-by-point raster scan to obtain the PL map. A short acquisition time of 10 ms/point (about 2 min for one PL mapping) is used to minimize the light exposure to the material and avoid the potential ion diffusion effect.

For the PL mapping during the recovery process, because the recovery process is very slow, in a time scale of many hours, the variation of the material during the 2 min PL mapping is minimal.

The carrier diffusion effect was practically non-existence, because the carrier decay time was in the order of 200 ns, while we probed the illumination effect well after the laser was turned off (at least 3–5 s). The illumination density was kept sufficiently low to avoid heating and photon-degradation^46,47^. The use of confocal PL mapping minimized the potential issue related to photon-recycling^48^.

### Time-resolved PL mapping

Excitation was provided by a dual-mode laser for pulsed and CW operation (Picoquant, LDH-P/D Series with PDL 828 driver), the wavelength for TRPL measurements was also 639 nm. TRPL mapping was conducted at ~2 × 10^12^ photons/(cm^2^ pulse) and 0.1 MHz frequency for the initial state. Then after a certain time of the local illumination via the CW excitation as that for the PL mapping, TRPL mapping was conducted at different recovery times. For mapping at low injection, decays at each pixel were fitted to a single exponential for simplicity. PL decay curves were measured at ~3.2 × 10^11^ photons/(cm^2^ pulse) and 0.1 MHz frequency at the illuminated site and ring.

### In situ optical transmission measurement

In situ optical transmission measurements were carried out using the bottom illumination lamp of the PL microscope stage. The bottom lamp provides a widefield illumination to the backside of the sample. The sample was placed on the sample stage and first illuminated locally with a laser beam from the top with ~12 μm beam size (4× objective lens) to generate the ring structure. To get a better spatial resolution, 100× was used to collect the transmission spectra of the bottom lamp at different spatial locations from the top side of the sample. The transmission spectra were collected at the brightest position of the ring and the center of the illuminated region. They are divided by the reference spectrum collected from the bare glass to get the final transmission of the sample.

## Supplementary information

Supplemental material
