# Supplementary material for: Photo-induced macro/mesoscopic scale ion displacement in mixed-halide perovskites: ring structures and ionic plasma oscillations
Source: Light Sci Appl. 2022 Sep 7;11:262. doi: 10.1038/s41377-022-00957-8 (PMC9448785; doi:10.1038/s41377-022-00957-8)
Supplement: Supplementary file 1 — Supplemental material [file 41377_2022_957_MOESM1_ESM.docx]

**SUPPLEMENTARY INFORMATION FOR**

**Photo-induced Macro/Mesoscopic Scale Ion Displacement in Mixed-Halide Perovskites: Ring Structure and Ionic Plasma Oscillations**

Xiaoxiao Sun^1,2,3,*^, Yong Zhang^4,*^, Weikun Ge^5^

^1^ Laboratory for Thin Films and Photovoltaics, Empa−Swiss Federal Laboratories for Materials Science and Technology, 8600 Duebendorf, Switzerland

^2^ Department of Information Technology and Electrical Engineering, ETH Zurich, 8093 Zurich, Switzerland

^3^ Helmholtz-Zentrum Dresden-Rossendorf, Institute of Ion Beam Physics and Materials Research, Dresden 01328, Germany

^4^ Department of Electrical and Computer Engineering, The University of North Carolina at Charlotte, Charlotte, NC 28223, USA

^5^ Department of Physics, Tsinghua University, Beijing 10084, People’s Republic of China

*Corresponding author. Email: [x.sun@hzdr.de](mailto:x.sun@hzdr.deh), [yong.zhang@uncc.edu](mailto:yong.zhang@uncc.edu)


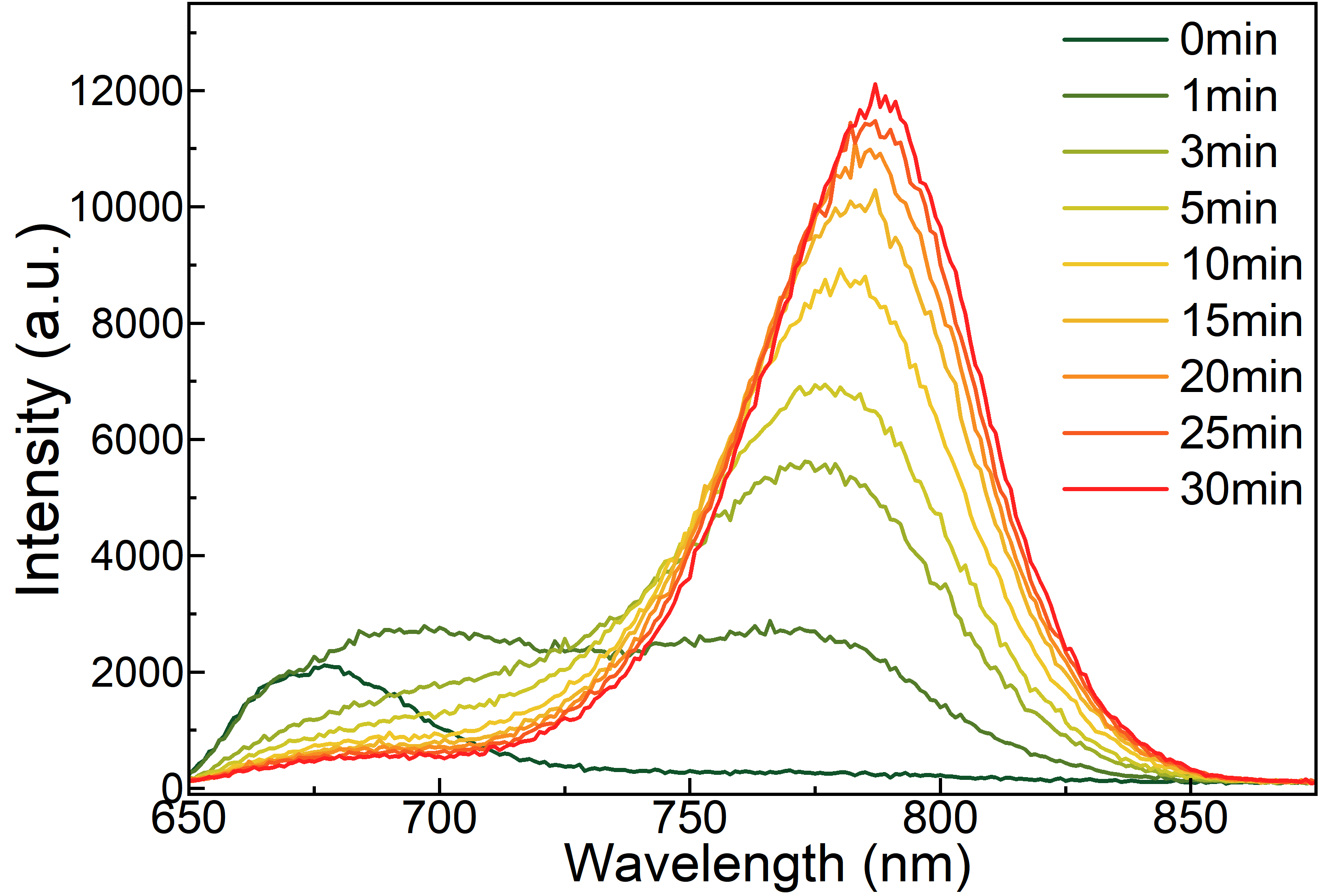


Fig. S1. PL spectra at illumination times over 30 min at 1 sun condition (0.1 W cm^-2^).


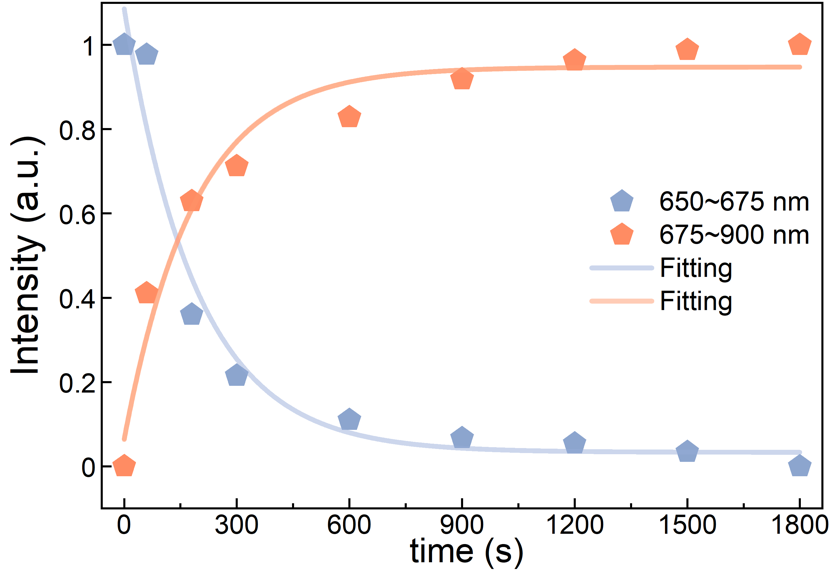


Fig. S2. The normalized integrated intensity below 675 nm and above 675 nm over time at the illumination site from Fig. S1.


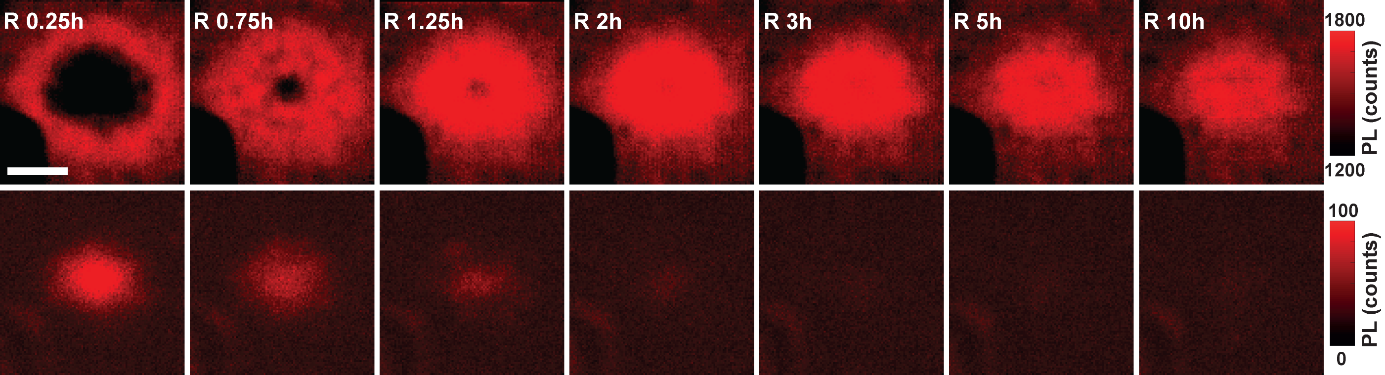


Fig. S3. PL emission mappings with detection wavelength at 670 nm (top row) and 790 nm (bottom row) during dark recovery from the back (glass side). The scale bar is 40 $\mu m$.


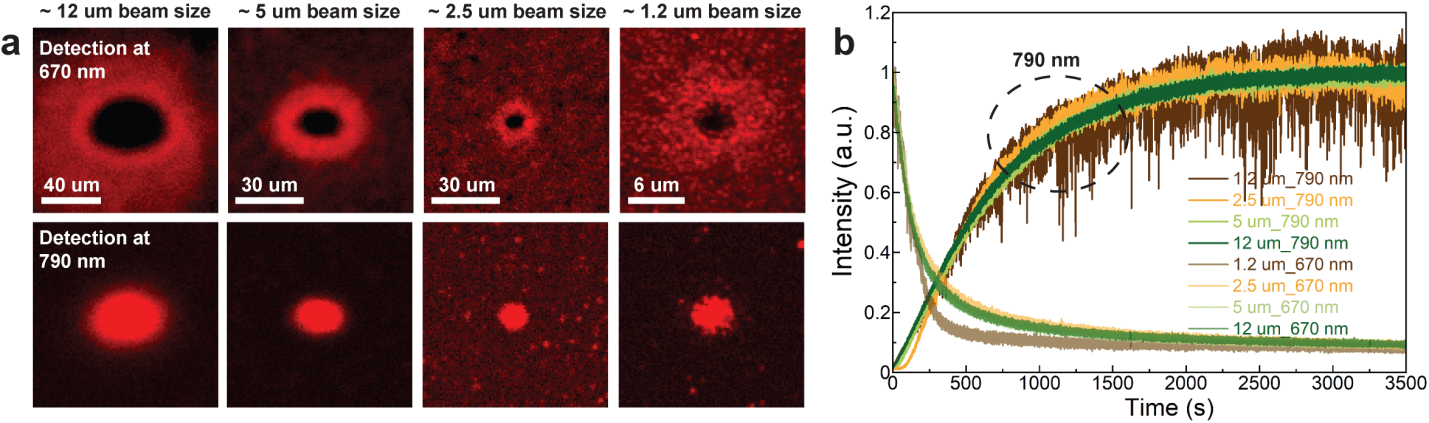


Fig. S4. (a) PL emission mapping at detection wavelengths of 670 nm and 790 nm after light illumination with different beam sizes. (b) Time map of 670 nm and 790 nm, respectively.


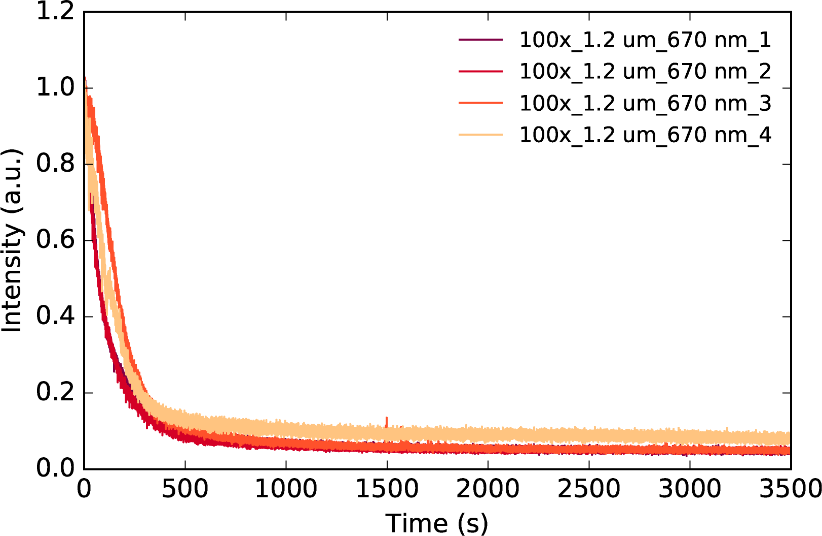


Fig. S5. Different sets of 670 nm time map with ~1.2 $\mu m$ illumination beam size.


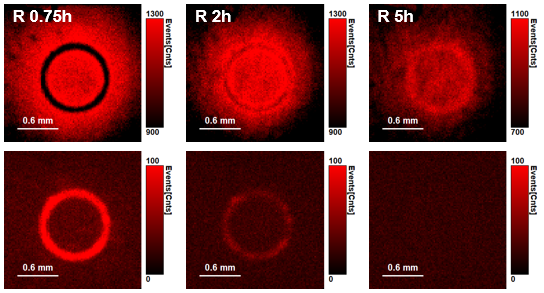


Fig. S6. The recovery process of light induced ion redistribution at different recovery times for 670 nm (top row) and 790 nm (bottom row). The beam size of 1 $\mathrm{mm}$ is over an annular aperture with a 1 $\mathrm{mm}$ diameter pinhole and a center obstruction target of 850 $\mu m$.


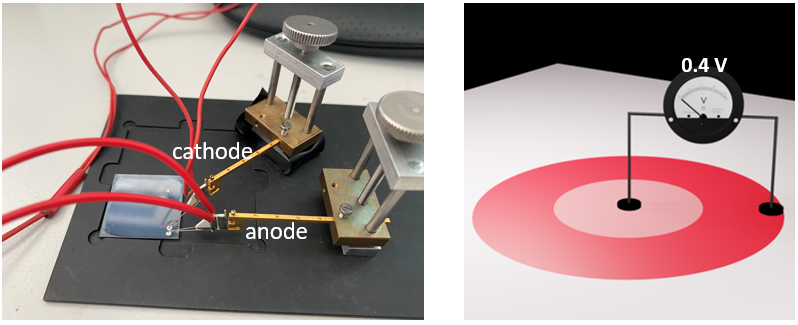


Fig. S7. The setup for the measurement of the electric potential difference between the illuminated area and ring area. The ion “segregation” phenomenon after local illumination led to a partial depletion of Br ions in the illuminated area, which was connected to the positive electrode, and the built-up of the Br ions at the ring which was connected to the negative electrode. To verify our theory, right after the sample was illuminated locally with a large beam size (~1 $\mathrm{mm}$), we quickly deposited a small drop of Ag paint at the center and a point very close to the ring, respectively, and used a voltmeter to measure the voltage change, as shown schematically above. The measurement yielded a value of 0.4 V. Despite being a primitive test, this result further supports our theory and suggests a potential application of the phenomenon, as a battery.


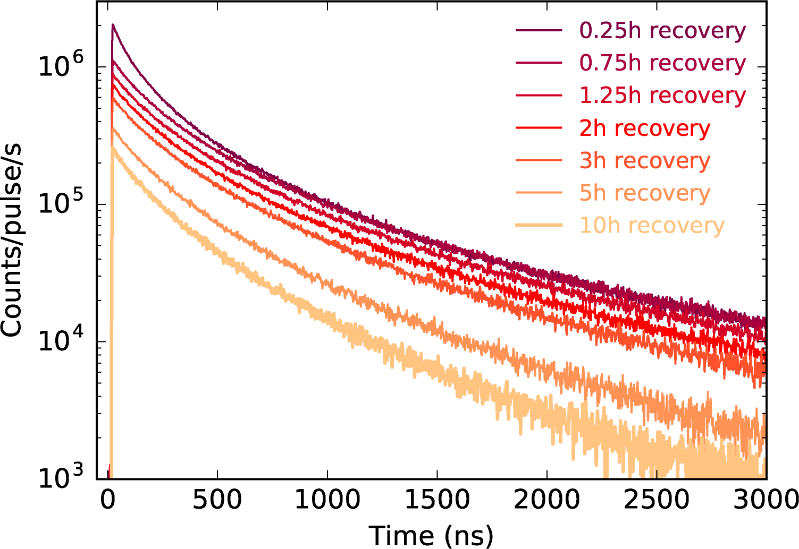


Fig. S8. The typical PL decay traces during dark recovery.


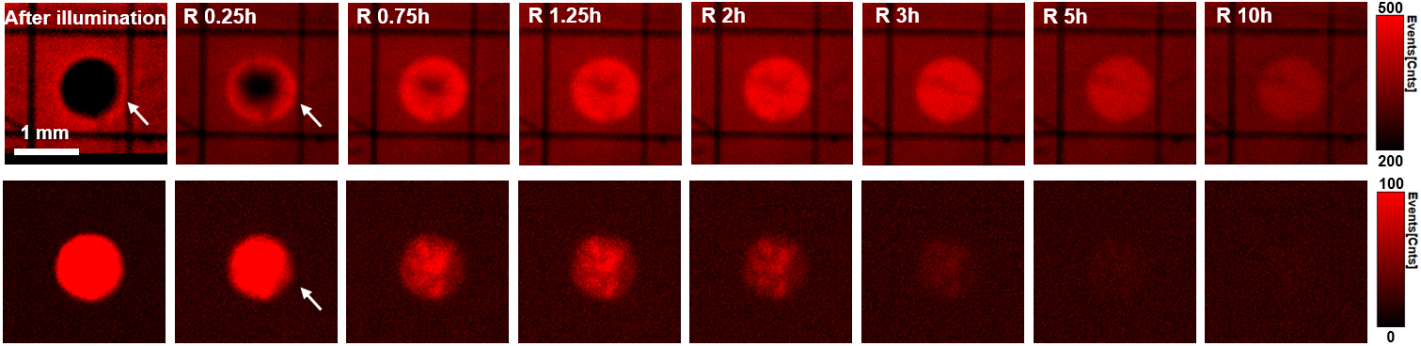


Fig. S9. PL emission mappings with detection wavelength at 670 nm (top row) and 790 nm (bottom row) after local illumination and during dark recovery in a pre-scribed area. The beam size is ~1 $\mathrm{mm},$ aligned with an annular aperture of 1 $\mathrm{mm}$ diameter pinhole. Using the same measurement condition as described in Fig. 3, we observe that the diffusion to the right was limited by the scribed line. During recovery, since the Br ions did not move out as much to the right due to the scribed line, the recovery of the right-hand side was faster than the left-hand side. This experiment supports the understanding that the Br ions are repelled from the illuminated area but the lateral motion is limited by the physical boundary of the sample.


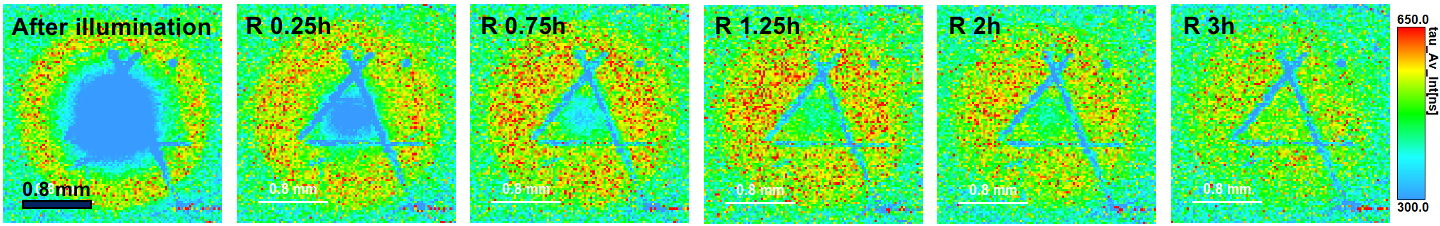


Fig. S10. TRPL mapping after local illumination and during dark recovery in a scribed triangle area. We attempt to scribe a triangle area smaller than the 1 $\mathrm{mm}$ beam size and perform the TRPL mapping after illuminating the area containing the triangular area. The measurement condition is the same as that of Fig. 4c. Inside the triangle area, the Br ions are pushed to the edge and corner of the triangle under light illumination, showing reduced PL lifetime. After 2h recovering, the Br ions slowly return to the center, with the corner region being slower than the closest edges. Outside the triangle, the Br ions are expelled from the illuminated region to form a ring, whereas they are blocked by the scribed lines during recovery. These findings indicate that the freed Br ions tend to reach the furthermost distance either determined by the Coulombic balancing force or the physical boundary, which further supports our conclusion that the motion of released Br ions from the illuminated site causes the “ion segregation”.


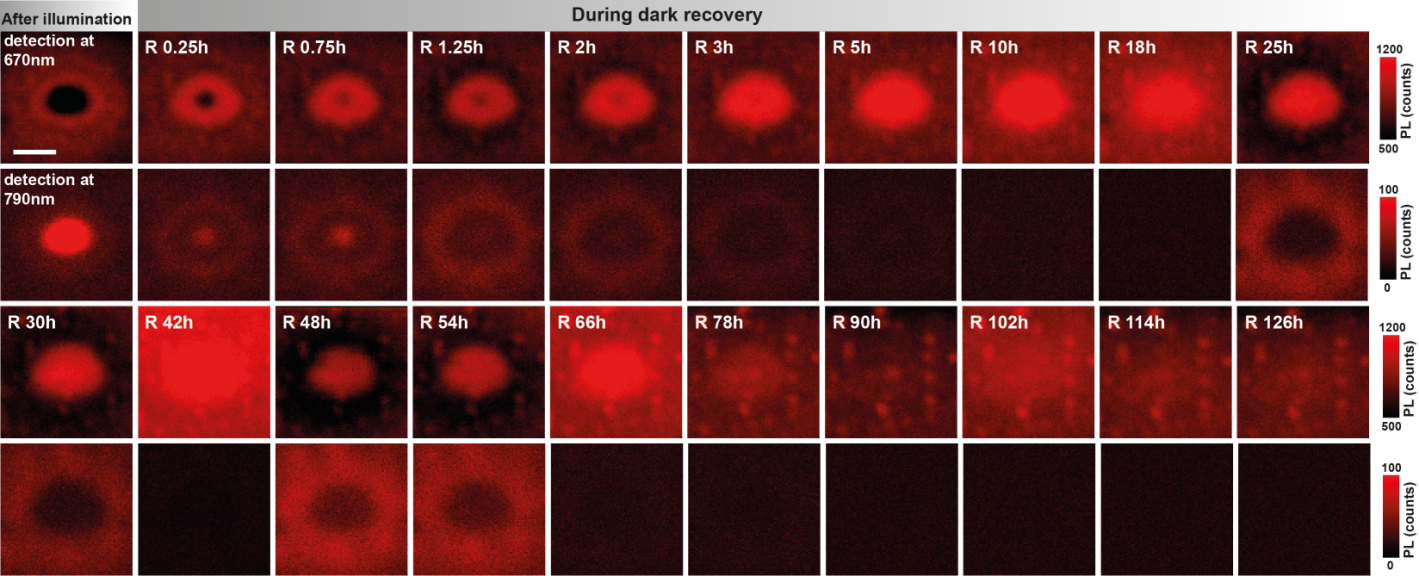


Fig. S11. A complete time series of Fig. 5: PL mapping after local illumination and at different recovery times for 670 nm (the first and third rows) and 790 nm (the second and fourth rows).


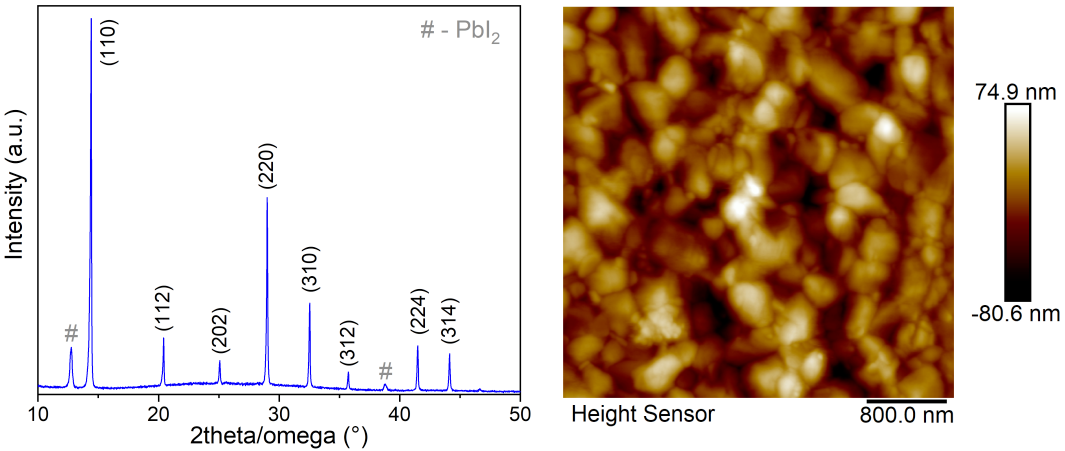


Fig. S12. X-ray diffraction spectrum and AFM image of MA_0.17_FA_0.83_Pb(I_0.5_Br_0.5_)_3_.

**Net ion charge distributions and free Br ion density calculations:**

*The calculation for the net ion charge distributions are given below:*


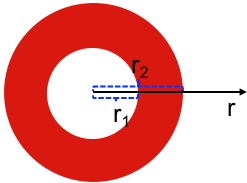


According to the Gauss theorem: $r<r_{1}$, $2\pi rhE\varepsilon=\pi{r^{2}hqN}_{1}$,

$E=\frac{{rqN}_{1}}{2\varepsilon}$,

$r_{1}<r<r_{2}$, $2\pi rhE\varepsilon=\pi(r_{2}^{2}-r^{2}){hqN}_{2}$,

$E=\frac{{qN}_{2}}{2\varepsilon}(\frac{r_{2}^{2}-r^{2}}{r}$),

where $E$ is the electric field, $\varepsilon$is the dielectric constant and $\varepsilon=$27.48$\varepsilon_{0}$, $h$ is the sample thickness.

$V_{build-in}=V\left( 0 \right)-V(r_{2})=\frac{{qN}_{1}}{4\varepsilon}r_{1}^{2}+\frac{{qN}_{2}}{2\varepsilon}[r_{2}^{2}ln(\frac{r_{2}}{r_{1}}\text{)-}\frac{r_{2}^{2}-r_{1}^{2}}{2}]$,

Applying the charge neutrality condition: $r_{1}^{2}N_{1}=(r_{2}^{2}-r_{1}^{2}$) $N_{2}$,

$V_{build-in}=\frac{{qN}_{2}}{2\varepsilon}r_{2}^{2}ln(\frac{r_{2}}{r_{1}}\text{)}$,

Based on the PL mapping results for the beam size of 1$\mathrm{mm}$ in **Fig. 3**, we have $r_{1}=$ 0.5 mm and $r_{2}=$ 1.1 mm. With the measured $V_{build-in}=$ 0.4 V, we have $N_{1}=$ 4.8 ×10^9^ cm^-3^ and $N_{2}=$1.25 ×10^9^ cm^-3^.

*Carrier generation rate* $G$ *is calculated using the following expression:*

$$G=\frac{P}{hv*A*d}$$

where $P$ is the incident pump power, $A$ is the area of the beam size and $d$ is the light penetration distance of the sample. Here we assume the generation efficiency to be 100%. The light penetration distance is defined as $1/\alpha$(absorption coefficient) for the pump wavelength used (639 nm). With the power density of 100 mW cm^-2^ (P/A) and pump penetration distance of 460 nm, $G$ is 6.8×10^21^ cm^-3^s^-1^.
